# Customized Flagelliform Spidroins Form Spider Silk-like Fibers at pH 8.0 with Outstanding Tensile Strength

**DOI:** 10.1021/acsbiomaterials.1c01354

**Published:** 2021-12-15

**Authors:** Xue Li, Xingmei Qi, Yu-ming Cai, Yuan Sun, Rui Wen, Rui Zhang, Jan Johansson, Qing Meng, Gefei Chen

**Affiliations:** † Department of Medical Ultrasound, Shanghai Tenth People’s Hospital, Ultrasound Research and Education Institute, Tongji University Cancer Center, Shanghai Engineering Research Center of Ultrasound Diagnosis and Treatment, Tongji University School of Medicine, 200092 Shanghai, China; ‡ Institute of Biological Sciences and Biotechnology, Donghua University, 201620 Shanghai, China; § The Jiangsu Key Laboratory of Infection and Immunity, Institutes of Biology and Medical Sciences, Soochow University, Suzhou 215123, China; ∥ Institute for Life Sciences, University of Southampton, SO17 1BJ Southampton, Hampshire, U.K.; ⊥ Department of Pulmonary Circulation, Shanghai Pulmonary Hospital, Tongji University School of Medicine, Shanghai 200433, China; # Department of Biosciences and Nutrition, Karolinska Institutet, 14157 Huddinge, Sweden

**Keywords:** flagelliform, customized FlSp, silk formation at pH 8.0, manual pulling, mechanical properties

## Abstract

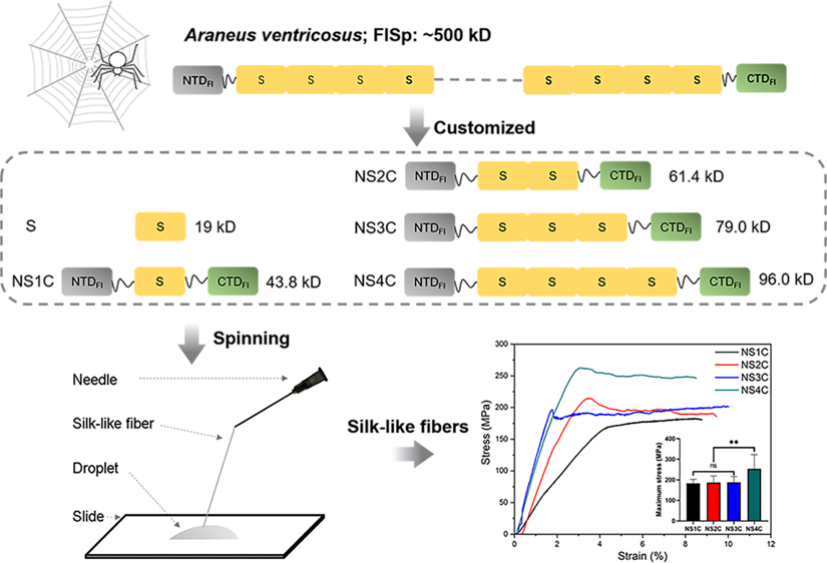

Spider flagelliform silk shows the best extensibility among various types of silk, but its biomimetic preparation has not been much studied. Herein, five customized flagelliform spidroins (FlSps: S and NTD_Fl_-Sn-CTD_Fl_, *n* = 1–4), in which the repetitive region (S) and N-/C- terminal domains (NTD_Fl_ and CTD_Fl_) are from the same spidroin and spider species, were produced recombinantly. The recombinant spidroins with terminal domains were able to form silk-like fibers with diameters of ∼5 μm by manual pulling at pH 8.0, where the secondary structure transformation occurred. The silk-like fibers from NTD_Fl_-S4-CTD_Fl_ showed the highest tensile strength (∼250 MPa), while those ones with 1–3 S broke at a similar stress (∼180 MPa), suggesting that increasing the amounts of the repetitive region can improve the tensile strength, but a certain threshold might need to be reached. This study shows successful preparation of flagelliform silk-like fibers with good mechanical properties, providing general insights into efficient biomimetic preparations of spider silks.

## Introduction

**Figure 1:**
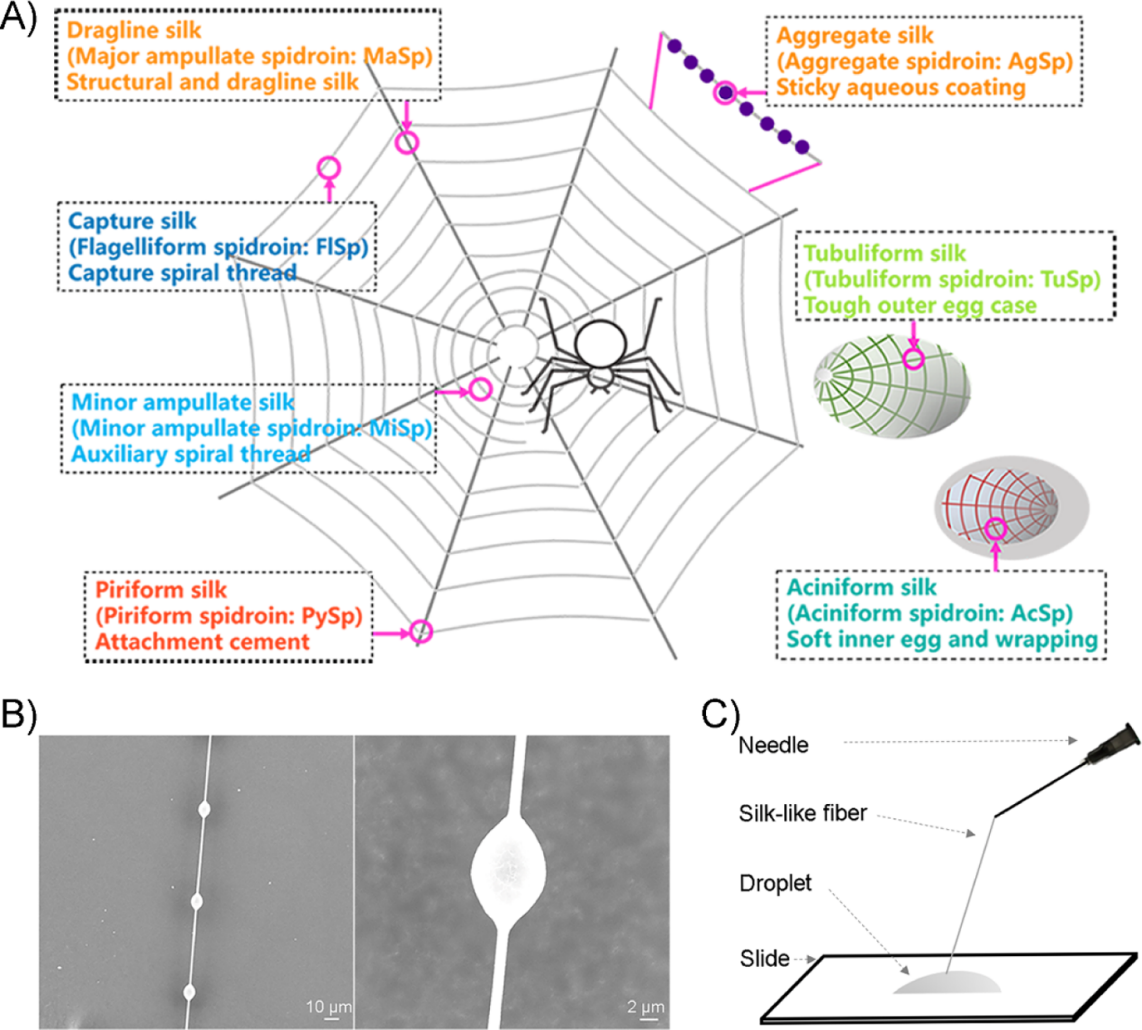
Orb-web spider silks and spinning strategy in this study. (A) Seven types of silk from orb-web spider, corresponding spidroins, and biological applications. (B) Scanning electron microscopy (SEM) images of native spider flagelliform silk generated in this study. The scale bars are 10 μm (left) and 2 μm (right), respectively. (C) Facile manual pulling strategy utilized for spider flagelliform silk-like fiber preparation from a recombinant protein droplet at pH 8.0.

Orb-weaving spiders produce up to seven types of silks with various mechanical properties for different biological purposes (Figure 1A).^1−3^ Spider silk as a biomaterial possesses outstanding mechanical properties,^1,4^ and excellent biocompatibility and biodegradability, holding great potential in biomedicine.^5,6^ Due to spiders’ innate territorial behavior and limited amounts of silks from a spider web, it is not realistic to obtain spider silk on a large scale via farming spiders. Recently, expressing trimmed spider silk proteins (spidroins) followed by silk-like fiber preparation has become the prevalent strategy for generating artificial spider silk-like fibers.^4,5^ Among different spider silks, dragline silk and flagelliform silk have attracted considerable attention attributed to their best tensile strength and extensibility, respectively.^2^

Each spider silk consists of a certain type of spidroins, which are responsible for specific mechanical properties and performance. For instance, dragline silk is made up of major ampullate spidroins (MaSps) with poly (A)_*n*_ motifs contributing to strength by forming β-sheet crystal structures,^2^ while flagelliform silk is composed of flagelliform spidroins (FlSps) that are rich in GPGGX (X is mainly for S or Y) motifs, resulting in intriguing extensibility, possibly through spring structures.^3,7^ The FlSp contains a large core repetitive region franked with NTD_Fl_ (FlSp N-terminal domain) and CTD_Fl_ (FlSp C-terminal domain). The core repetitive region is made up of 11 repetitive units that are built by iterations of the GPGGX, GGX (X is mainly for S, Y, or A), and hydrophobic spacer motifs.^7^ These functional motifs confer flagelliform silk the best extensibility among other spider silks as well as excellent strength, and it has been shown that the GPGGX, GGX, and spacer motifs contribute to extensibility, toughness, and strength, respectively, in synthetic silk-like fibers.^8−10^ While several studies have looked into the preparation of artificial flagelliform silk, only a few chimeric FlSp/MaSp spidroins were successfully constructed for producing spider silk-like fibers,^11−14^ which might be due to the fact that FlSp contains highly repetitive units rich in Gly and Pro, making it difficult to be expressed in exogenous systems. Meanwhile, the mechanism of molecular self-assembly or the structural/functional investigations of FlSp remains largely missing, in particular the terminal domains, NTD_Fl_ and CTD_Fl_. NTD and CTD are highly conserved with regard to the tertiary structure, where NTD adopts a five α-helix structure that dimerizes into an antiparallel dimer at low pH^15−23^ and CTD is a parallel dimer with α-helical bundles.^24−28^ NTD allows spidroin storage in silk gland and progressively forms stable dimers in a pH-dependent manner.^18,21^ CTD, on the other hand, can unfold and assemble into amyloid-like fibrils which can then trigger silk formation or regulate silk formation and improve mechanical properties.^25,27,28^ To obtain high-quality artificial spider silk-like fibers, it is probably essential to include both NTD and CTD. Indeed, some designed spidroins, such as mini-MaSp^29,30^ and mini-AcSp (aciniform spidroin),^31^ have been recombinantly generated and spun into spider silk-like fibers with decent mechanical properties without using an organic solvent. Furthermore, the terminal domains, in particular the CTD from different spidroins, display various roles in the silk formation process; for example, CTD from MaSp is important for the correct structure of nanofibers,^32^ while AcSp CTD is important for maintaining silk mechanical properties and uniquely modulates silk fiber properties.^24,33^ Hence, customized spidroins with the repetitive regions fused with its natural terminal domains might benefit the biomimetic preparation of specific type of silk-like fibers.

Here, five customized spidroins [S, and NTD_Fl_-Sn-CTD_Fl_, (NTSnCT) with *n* = 1–4] were generated from the full-length FlSp of spider *Araneus ventricosus*, that is, the NTD_Fl_, repetitive region S, and CTD_Fl_ are all from the same spidroin, FlSp, and the same spider species (*A. ventricosus*). The customized spidroins were recombinantly produced in *Escherichia coli* and spun into spider silk-like fibers directly from physiological buffer at pH 8.0. Furthermore, mechanical properties and secondary structure transformation from soluble protein solutions to solid silk-like fibers were investigated by tensile testing, circular dichroism (CD) spectroscopy, and attenuated total reflection Fourier-transform infrared (ATR-FTIR) spectroscopy. This study has prepared artificial spider flagelliform silk-like fibers via a facile manual-method which simultaneously covers FlSp-derived repetitive region and teriminal domains, and the results contribute to the development of novel potential biomaterials.

## Materials and Methods

### Plasmid Construction

**Figure 2:**
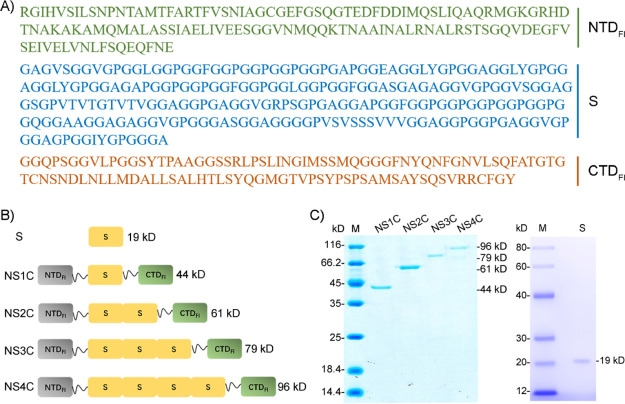
Architecture of different customized FlSps and SDS-PAGE analysis of recombinant preparations. (A) Amino acid sequences of the Flag repetitive region S, NTD_Fl_, and CTD_Fl_ used in this study. (B) Architecture of customized FlSps (S, NS1C, NS2C, NS3C, and NS4C). NTD_Fl_ and CTD_Fl_ indicate N- and C- terminal domains from *A. ventricosus* flagelliform spidroin, respectively. S stands for the repetitive region trimmed from *A. ventricosus* flagelliform spidroin. (C) SDS-PAGE analysis of the purified customized FlSps—S (right), NS1C, NS2C, NS3C, and NS4C (left). Lane M is the protein marker.

Five plasmids that code for *A. ventricosus* FlSp spidroins, S, NS1C, NS2C, NS3C, and NS4C, were constructed, resulting in customized spidroins with different numbers of repetitive region S optionally flanked with *A. ventricosus* FlSp terminal domains—NTD_Fl_ and CTD_Fl_. The NTD_Fl_, repetitive region S and CTD_Fl_ amino acid sequences are shown in Figure 2. The DNA sequences for *A. ventricosus* FlSp NTD, CTD, and S were obtained through screening the previously constructed *A. ventricosus* genomic library^34^ and further confirmed by phylogenetic analysis. Polymerase chain reaction primers used in this study are listed in Table S1. The gene fragment encoding the repetitive region S was optimized for efficient expression in *E. coli* BL21. All the constructs were confirmed by sequencing.

### Recombinant Customized FlSp Spidroin Preparation

Customized FlSps were expressed in *E. coli* BL21. A single colony of each construct was cultured in 10 mL of LB medium with 100 μg/mL ampicillin at 37 °C overnight and then transferred to 1 L of fresh LB medium with 100 μg/mL ampicillin. When OD_600_ reached 0.6–0.8, the incubation temperature was cooled down to 25 °C and 0.3 mmol/L (final concentration) isopropyl β-*d*-1-thiogalactopyranoside was added. Cells were incubated for another 16 h and collected by centrifugation at 4500 rpm for 15 min and further resuspended in 30 mL of 20 mmol/L Tris pH 8.0. The cells were lysed by a pressure homogenizer JN-3000 plus (JNBIO) under 1 200 bar. The supernatant was collected after centrifuged at 10 000 rpm (4 °C) for 40 min and applied to Ni-NTA column, followed by a 1 h incubation. The column was washed with 20 mmol/L Tris pH 8.0 containing 5 mmol/L imidazole, and target proteins were subsequently eluted out by 20 mmol/L Tris pH8.0 with 100 mmol/L imidazole that was eventually removed by dialysis against 20 mmol/L Tris pH 8.0.

### CD Spectroscopy

Customized FlSps with a final concentration of 0.5 mg/mL in 20 mmol/L phosphate buffer (pH 7.5, 6.5, and 5.5) were subjected to CD measurements. For each protein, the samples at different pHs were diluted from the same protein stock to maintain the same concentration. CD spectra were recorded in 1 mm path length quartz cuvettes at 25 °C from 260 to 190 nm in a J-810 spectropolarimeter (JASCO, Japan) at room temperature, and the main parameters were as follows: wavelength step 0.5 nm, response time 1 s, and bandwidth 1 nm. The individual spectrum shown for each was the average from three continuous scans with background (20 mmol/L phosphate buffer) subtracted.

### Spider Silk-like Fiber Generation

As previously reported,^35^ spider silk-like fibers were manually drawn by hand (manual pulling) from recombinant customized FlSp solutions in 20 mmol/L Tris pH 8.0 (∼1 mg/mL) with a syringe needle (26G) at room temperature, as shown in Figure 1C. Briefly, 100 μL of recombinant spidroin solution was dropped onto a glass slide, and spider silk-like fibers were pulled from the solution with a continuous speed of ∼10 mm/s.

### Scanning Electron Microscopy

The native flagelliform silk collected from the web of wild *A. ventricosus* and artificial spider silk-like fibers spun from customized FlSp spidroins were fixed onto SEM plates and coated with gold for 45 s. Images were taken by a SEM system (Phenom-WorldBV) with 10 kV acceleration voltage at room temperature.

### Mechanical Property Test

Mechanical properties of silk-like fibers were tested with UTM T150 (Agilent). Prior to mechanical tests, the uniform thickness of the individual silk-like fiber was confirmed via light microscopy, during which the diameters of different silk-like fibers were measured. For strain–stress measurements, silk-like fibers were fixed into a 1 cm frame and mechanical test was performed with starting load strength being 750 μN at 20 °C and 50% humidity. For each type of the silk-like fibers, 10 samples were supposed to be tested; however, some of them were broken before applying the starting load strength, which subsequently lead to seven (for NS1C, NS2C, and NS4C) or nine (for NS3C) valid tests for each type of silk-like fibers. Statistical analysis was conducted by using one-way analysis of variance (ANOVA), followed by Tukey’s post-hoc test for multiple comparisons. *p < 0.05, **p < 0.01.

### ATR-FTIR Analysis

Nicolet 6700 spectroscopy system with ATR fitting (Thermo Fisher) was used to record ATR-FTIR spectra of silk-like fibers from 600 to 4 000 cm^–1^ at room temperature.^12,29,31^ Sixty-four scans were collected for each spectrum with a resolution of 4 cm^–1^. The background spectrum of a blank was subtracted. Each type of the silk-like fiber was analyzed three times (for each type, a mass of silk-like fibers was fixed for recording ATR-FTIR spectra, as a single silk-like fiber was not suitable for ATR-FTIR measurement). The transmittance (*T*) was transformed into absorbance (*A*) according to the function *A* = 2–log(% *T*). Spectral decomposition of the amide I region ranging from 1 600 to 1 700 cm^–1^ was performed using the peak-fit program (Cranes Software International Ltd.), and secondary structure composition was subsequently evaluated. A seven-point Savitsky-Golay second-derivative function was implemented to separate overlapping bands in the amide I region, which was followed by curve-fitting (Gaussian peak shape). Before curve fitting, a linear baseline was subtracted in the amide I band from 1600 to 1700 cm^–1^. Different secondary structures present distinct characteristic peaks in the amide I region (R^2^ values are nearly 1); characteristic peak near 1 639 cm^–1^ is for random coil, 1 656 cm^–1^ for α-helix, 1 669 cm^–1^ for β-sheet, and 1 684 and 1 698 cm^–1^ for β-turn.^31^

## Results

### Preparation of Customized *A. ventricosus* FlSps

The FlSp repetitive region (S) containing typical FlSp motifs was selected to fuse with its native NTD_Fl_ and CTD_Fl_ (Figure 2A,B). The repetitive region S is rich in Gly (55%) (Figure S1A), and the Gly content increased when more repetitive region S were included. With 10% of Ile, Leu, and Val, the repetitive region S showed several hydrophobic stretches; however, hydrophilic patches were also observed (Figure S1B). The customized *A. ventricosus* FlSps containing different numbers of repetitive region S with and without NTD_Fl_ and CTD_Fl_ were designated as S, NS1C, NS2C, NS3C, and NS4C (Figure 2B). These five customized FlSps were successfully expressed in *E.coli* and mainly present in the soluble fraction after cell lysis and centrifugation. After Ni-NTA affinity column purification, the purity of customized FlSps reached 90% as judged by sodium dodecyl sulfate-polyacrylamide gel electrophoresis (SDS-PAGE) (Figure 2C). The final yields for S, NS1C, NS2C, NS3C, and NS4C were ∼10, ∼70, ∼66, ∼25, and ∼13 mg/L, respectively, indicating that the terminal domains can promote the recombinant preparation, whereas the length of the repetitive region is inversely proportional to the final yield.

### Secondary Structure of Customized FlSps at Different pHs

**Figure 3:**
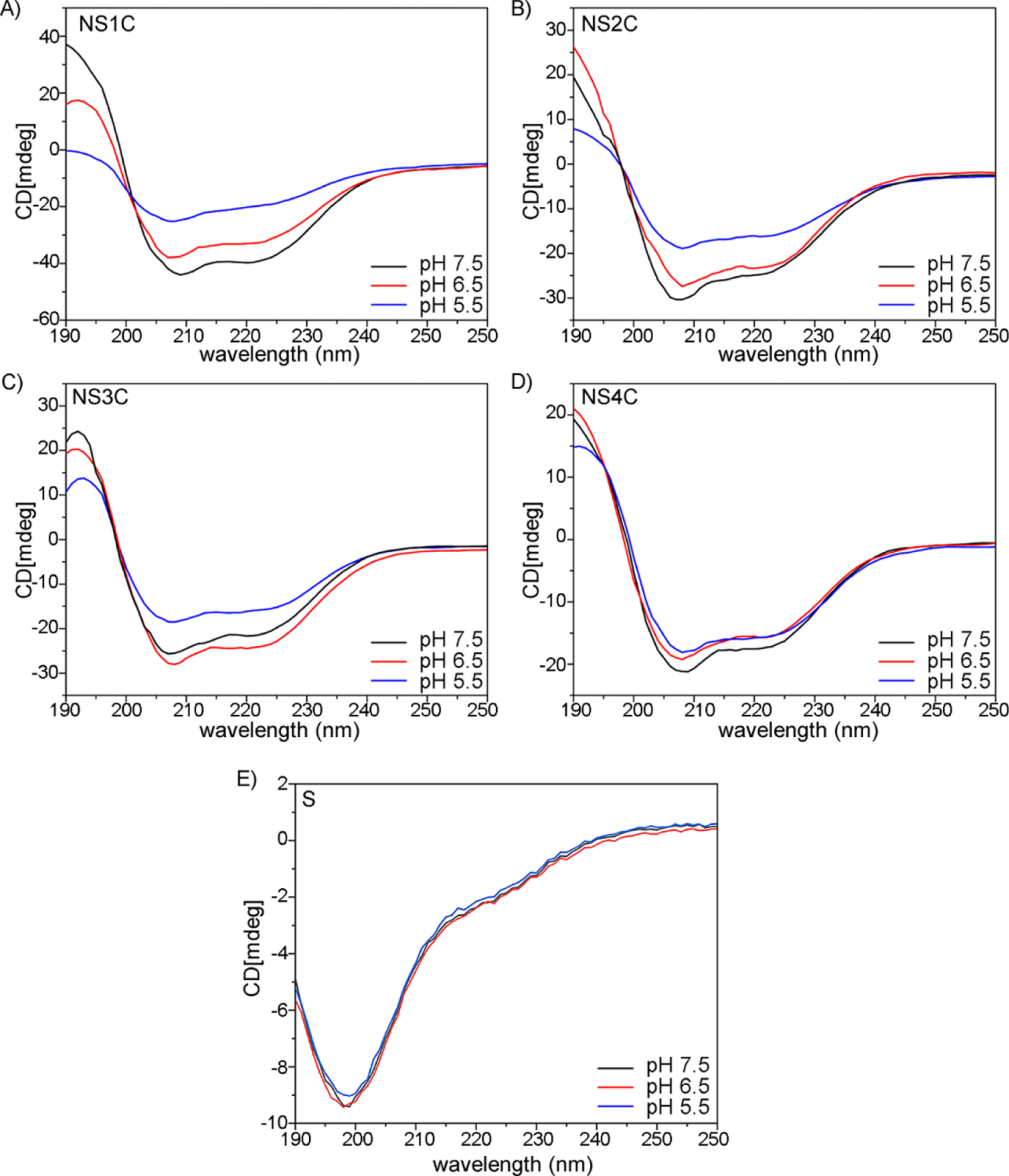
CD spectra of customized FlSps under different pHs. CD spectra of customized FlSps in 20 mM phosphate at different pHs (7.5, 6.5, and 5.5) were recorded at room temperature. (A) NS1C. (B) NS1C. (C) NS3C. (D) NS4C. (E) S. The *Y* axis is given by mdeg.

As pH gradient is necessary for spider silk formation, hence, we investigated pH effects on the potential secondary structure transformation of customized FlSps by CD spectroscopy at different pHs (7.5, 6.5, and 5.5, Figure 3). At pH 7.5, the recombinant repetitive region S mainly adopted random coil and partially helical structures, which did not display obvious changes when the pH decreased to 5.5 (Figure 3E). All the other customized FlSps (NS1C, NS2C, NS3C, and NS4C) showed overall α-helix conformation, indicated by two typical negative peaks at 208 and 222 nm (Figure 3A–D). However, the strong α-helix signal could be probably derived from the NTD_Fl_ and CTD_Fl_ domains because NTD and CTD domains from other spidroins mainly consist of five α-helixes and these two domains are highly conserved among different types of spidroin.^18,19,21,24,25,27,28^ With pH decreased to 5.5, the overall shapes of the CD spectra of NS1C, NS2C, and NS3C were not affected significantly, although lower amplitude was observed (Figure 3A–C), which might result from the decline of the amount of protein in solution induced by acidification. Interestingly, pH decrease did not substantially influence the amplitude of NS4C (Figure 3D), which could be probably due to that NS4C predominantly consists of the core repetitive region (four S regions) which maintained its secondary structure at different pHs (Figure 3E).

### Spider Silk-like Fiber Preparation from Customized FlSps and Characterization

**Figure 4:**
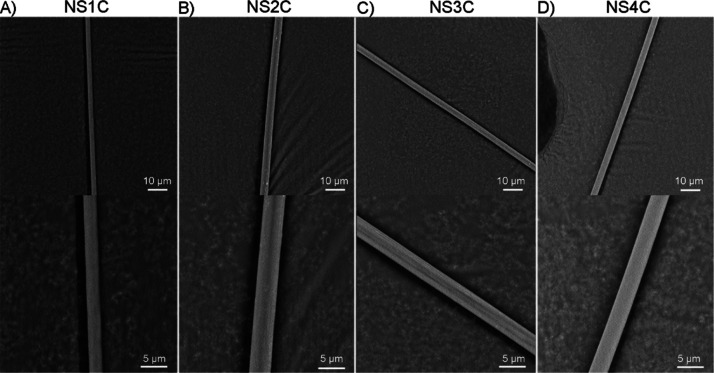
SEM observation of different silk-like fibers from customized FlSps. Spider silk-like fibers were prepared via the manual-pulling method and observed under SEM. (A) NS1C. (B) NS1C. (C) NS3C. (D) NS4C. The scale bars are 10 μm (upper panel) and 2 μm (lower panel), respectively.

To prepare flagelliform silk-like fibers from the recombinant customized FlSps, a straightforward spinning method was pursued, by which spider silk-like fibers were directly generated by manual pulling from different customized FlSp solutions with a syringe needle (Figure 1C). The recombinant single repetitive region S was soluble and could not assemble into silk-like fibers, at least by direct manual pulling from 20 mmol/L Tris pH 8.0. Interestingly, all the other customized FlSps with the terminal domains were able to form long solid silk-like fibers (>10 cm) as “spun” by manual pulling at pH 8.0, suggesting that the terminal domains are essential for silk formation under the spinning conditions. Neutral pHs are used for spidroin storage in the spider silk gland, and acidic pHs are essential for spider silk protein solidification.^28^ The observations here suggest that pH is probably not the only determinant for solid silk formation from soluble spidroins. Compared to native spider flagelliform silk (diameter as ∼2 μm), all silk-like fibers pulled from NS1C, NS2C, NS3C, and NS4C droplets displayed similar diameters (2–5 μm) (Figures 1B and 4C). These silk-like fibers all presented smooth surface morphology and no obvious grooves or pores were observed under SEM (Figure 4).

### Mechanical Properties and Secondary Structures of Silk-like Fibers

**Figure 5:**
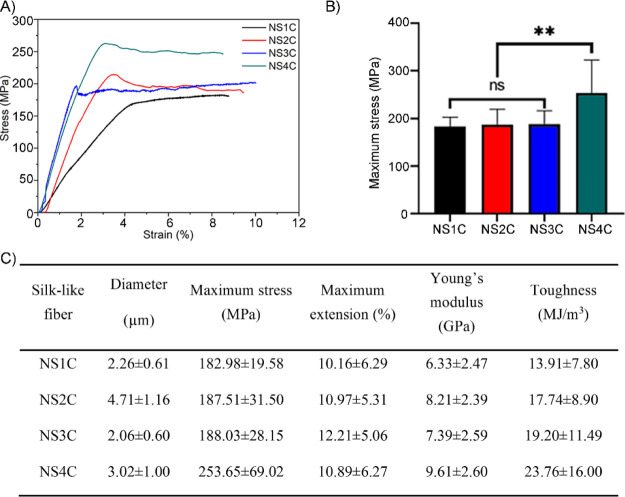
Mechanical property of silk-like fibers from the customized FlSps. (A) Stress–strain curves of silk-like fibers from NS1C, NS2C, NS3C, and NS4C. (B) Tensile strength comparison, where ns is for no significant difference and ** for *p* < 0.01. (C) Mechanical properties of silk-like fibers. The mean values were calculated from independent tests of each silk type, and data were shown as mean ± standard deviation.

Spider silk-like fibers derived from customized FlSps (NS1C, NS2C, NS3C, and NS4C) were subjected to mechanical property assessment at room temperature with 50% humidity. The mechanical properties are shown in Figures 5 and S2. All the silk-like fibers showed outstanding tensile strength (>180 MPa, Figure 5A,B). The largest protein studied here, NS4C fibers broke at the highest stress, 254 MPa on average (Figure 5A,B). In addition, the silk-like fiber from the NS4C fiber also showed the best toughness (24 MJ/m^3^) and Young’s modulus (10 GPa) (Figure 5C), suggesting a positive relationship between the molecular weight (the number of repetitive region S) and the corresponding mechanical properties. However, there were no significant differences of the maximum tensile strength between NS1C, NS2C, and NS3C (Figure 5B), indicating there is probably a molecular weight threshold for positive correlations between molecular weight and mechanical properties. In terms of extensibility, no obvious differences were noticed among the different silk-like fibers, and the average extension for all the silk-like fibers reached 10–12% (Figure 5C), which is considerably lower compared to the native spider flagelliform silks (>200%).^7^

**Figure 6:**
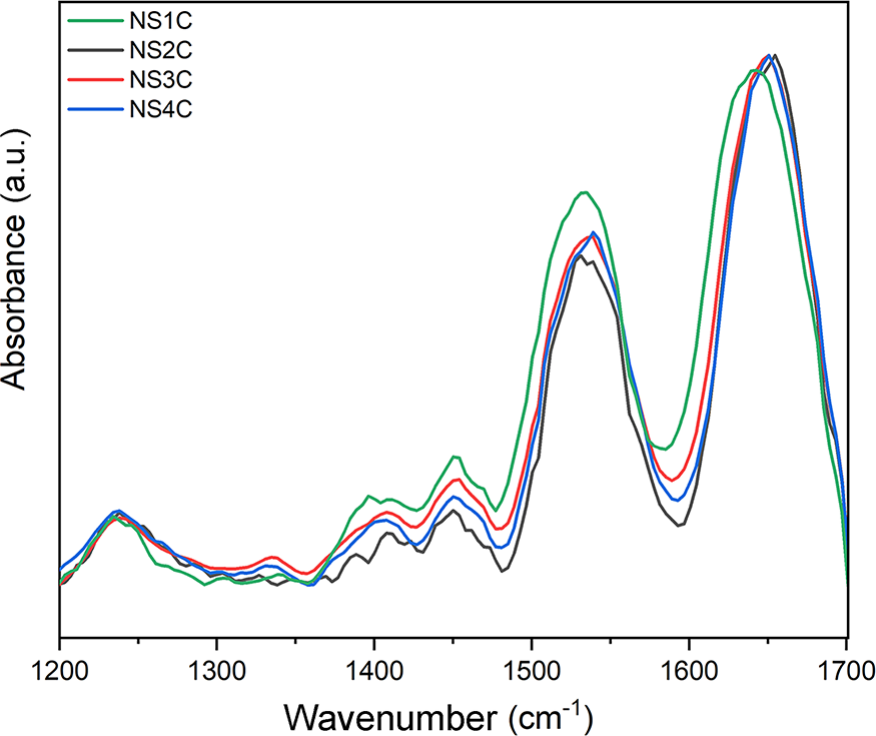
Secondary structure evaluation of silk-like fibers from customized FlSps by ATR-FTIR.

Generally, during spider silk formation, spidroins will experience structure transformation from disordered and partly helical structures to β-sheets and “amorphous” conformations.^36^ Here, the secondary structures of spider silk-like fibers were explored with ATR-FTIR. Despite roughly consistent ATR-FTIR spectra of silk-like fibers from NS1C, NS2C, NS3C, and NS4C (Figure 6), the ATR-FTIR spectrum of NS1C silk-like fiber presented a slightly left-shift peak near 1630 cm^–1^ compared to other types of silk-like fibers, which suggests that it contains different secondary structure compositions, for example, more β-sheet structure.^29^ Furthermore, spectral decomposition of the amide I region from 1600 to 1700 cm^–1^ was performed with peak-fit software to evaluate the secondary structure composition (Figure S3). Compared to other types of silk-like fiber, NS1C silk-like fiber adopted more β-sheet structure (43%) but less β-turn conformation (27%) (Table S2). Although NS1C silk-like fibers contained the highest amount of β-sheet structure, it did not show a relative better tensile strength compared to other silk-like fibers. The silk-like fibers from NS4C broke at the highest stress point, but the secondary structure profile evaluated from ATR-FTIR spectrum was similar to that of silk-like fibers from NS2C, NS3C (Table S2), suggesting that the mechanical properties cannot be perfectly reflected by secondary structure composition and there might be other determinants. The results here support that recombinant spidroins with larger molecule weight generally were leading to silk-like fibers with better strength.^31,37^

## Discussion

In this study, five customized FlSps, S, NS1C, NS2C, NS3C, and NS4C, with terminal domains and repetitive region from the same spidroin and spider species, were successfully produced recombinantly. The repetitive region S was soluble and could not assemble into a silk-like fiber induced by manual pulling at pH 8.0, while all the other customized FlSps formed spider silk-like fibers with outstanding strength and decent extensibility.

Spider flagelliform silk owns the best extensibility among different types of spider silk, which could have unique application potential, for example, cardiac tissue engineering applications.^38^ The repetitive region and terminal domains of spidroin function differently,^8,14,18,21,27,28^ therefore, in this study besides the single repetitive region S, four customized FlSps containing NTD_Fl_, S, and CTD_Fl_ which are all from *A. ventricosus* FlSp were generated for biomimetically preparing spider flagelliform silk-like fibers. These recombinant customized FlSps all showed considerable expression yield, even though the molecular weight of the largest customized FlSp NS4C reached 96 kDa (Figure 2). All the recombinant customized FlSps were in the soluble fraction during purification, and the purified proteins also showed excellent solubility. For the customized FlSps with NTD_Fl_, high solubility is expected because NTD is extremely soluble and has been implemented to produce membrane proteins, enzymes, and hydrophobic amyloid prone peptides.^39−44^ Interestingly and surprisingly, the recombinant single repetitive region S without NTD_Fl_ and CTD_Fl_ was also present in the soluble fraction. It has been shown that repetitive regions from MaSp,^37^ MiSp,^26^ TuSp (tubuliform spidroin),^45^ and PySp^46^ were expressed in inclusion body and needed to undergo denaturing and refolding processes, and the results here suggest that different repetitive regions from various spidroins have different biochemical properties.

Without NTD_Fl_ and CTD_Fl_, recombinant S did not self-assemble into silk-like fibers by direct pulling at pH 8.0, indicating that terminal domains, NTD_Fl_ and CTD_Fl_, are essential for spidroin assembly. However, this study does not exclude the possibility that the single repetitive region S could form fibers under some other specific conditions. The detailed structure/function correlations of N-/C-terminal domains of FlSp are poorly understood, although they are conserved between different spidroins.^4^ The C-terminal domains from MaSp and AcSp both adopt an identical α-helical structure and can fold into dimer conformations, but they play distinct functional roles. For instance, a recombinant designed spidroin MaSp [(AQ)_12_NR3] containing MaSp CTD formed regular well-defined fibers, whereas (AQ)_24_ only without the CTD formed random aggregates;^27^ for the CTD from AcSp, it mainly contributes to mechanical properties of synthetic fibers, and no difference was found between the macro morphologies of fibers with or without CTD.^24,25^ The repetitive domain S mainly maintained random coil and partially helical conformations (Figure 3E), which is quite different from that of other spidroin repetitive regions, for example, AcSp repetitive domain contains seven^47^ or five α-helixes^48^ depending on the spider species, while TuSp repetitive domain has six α-helixes.^49^ This suggests that the silk formation mechanism of FlSp can be different from other spidroins.^47^ Hence, for customized spidroin designing, the NTD, repetitive region, and CTD from the same spidroin and even the same spider species can benefit a specific type of biomimetic silk-like fiber production.

Chimeric mini-spidroins have shown the capability to be spun into silk-like fibers by wet spinning or hand-pulling method at different pHs. NT_MaSp_-2Rep-CT_MiSp_ was spun to silk-like fibers at pH 5.0, where high protein concentration was used (100–500 mg/mL).^29^ Similarly, N_MaSp2_-R7-C_MaSp1_ at 300 mg/mL was spun into silk-like fibers at pH 5.0.^30^ Chimeric spidroins NT_MaSp_-Rep-CT_MiSp_ at 100–300 mg/mL were spun into silk-like fibers from pH 2.0 to 11.^50^ Chimeric spidroins W_AcSp_2C_MaSp_4CT_MaSp_ and W_AcSp_2C_MaSp_8CT_MaSp_ formed silk-like fibers by hand-pulling at pH 7.5 at rather low concentration (0.4 mg/mL). Recently, silk-like fibers were pulled manually from NT_MaSp_R_MaSp/FlSp_nCT_MaSp_ (n indicates the number of R in combination of MaSp and FlSp repeats)^11^ and from NT_MaSp_W_AcSp_nCT_MiSp_ at concentrations of (0.8–1.0 mg/mL) at pH 8.0.^31^ It is also interesting that silk-like fibers can be drawn from only two repetitive regions from AcSp, W_AcSp_2 (20–200 μmol/L) at pH 7.5.^48^ In this study, the customized FlSp with terminal domains here could form silk-like fibers at rather low concentrations (∼1 mg/mL) via manual pulling at pH 8.0 (Figure 4). Taken together, the above phenomenon suggests that pH is probably not the sole determinant, but there are other factors that can affect spidroin-silk transformation.

The silk-like fibers from customized FlSps, NS1C, NS2C, NS3C, and NS4C displayed excellent tensile strength, ranging from 180 to 250 MPa, and reasonable extensibility of 10–12% (Figure 5C). Silk-like fibers pulled from chimeric spidroin NT_MaSp_W_AcSp_nCT_MiSp_ (*n* = 1–4) with the manual pulling method presented a tensile strength range from 51 to 245 MPa,^31^ while silk-like fibers from NT_MaSp_W_AcSp_1CT_MiSp_ (45.8 kDa) presented ultimate tensile strength of 51 MPa, extensibility of ∼12%, a Young’s modulus of 2.8 GPa, and a toughness of 4.8 MJ/m^3^. Silk-like fibers from the smallest customized FlSp NS1C (44 kDa) in this study broke at a maximum strength of 180 MPa, a maximum extensibility of 10%, a high Young’s modulus of 6 GPa, and a toughness of 14 MJ/m^3^ (Figure 5C). The mechanical properties of silk-like fibers from customized FlSps are also comparable to synthetic silk-like fibers produced via wet-spinning strategy. Recombinant fibers from chimeric spidroins NT_MaSp_-Rep_MaSp_-CT_MiSp_ (44 kDa) showed a tensile strength of about 50 MPa but a high extensibility of ∼90%.^50^ The tensile strength of synthetic fibers from chimeric spidroins NT_MaSp_-2Rep_MaSp_-CT_MiSp_ (33 kDa) reached 162 MPa and these silk fibers had an extensibility of 37%.^29^ The customized FlSp silk-like fibers in the current study revealed an outstanding tensile strength comparable to the wet-spinning silk-like fibers, but a relatively lower extensibility, which was not expected. As spider flagelliform silk is the most elastic among different types of spider silk, contributed by its spidroin amino acid composition, these silk-like fibers spun from different FlSp versions would be expected to have an excellent extensibility. This discrepancy can probably result from the spinning method that is very different from the spider spinning system and the wet-spinning method used in literature where shear force, spinning rate control, highly concentrated dope, and coagulating bath (normally at low pH) were all involved.^29^ On the other hand, the amino acid sequence of repetitive S selected and the limited size of recombinant customized FlSps are also possible explanations for the weak extensibility compared to the native fibers, as the size of spidroin can affect fibril properties.^31,37^ The main secondary structures of the customized FlSps in phosphate buffer were α-helix and random coil, while the dominant secondary structures of the silk-like fibers were β-sheet and β-turn. These results are consistent with the previous study, where the solid silk formation is accompanied by structure transformation of spidroins.^1^ However, the secondary structure content of the flagelliform silk-like fibers is significantly different from the native flagelliform silk,^9^ which may explain the difference in mechanical properties of the recombinant flagelliform silk-like fibers in this study. For future artificial silk-like fiber preparation, the spinning process and/or the protein constructs should promote the final product to recapitulate corresponding native silks to obtain high-performance artificial fibers.

## Summary

Recombinant spidroin-like proteins are normally dissolved in an organic solvent to make dopes for spinning artificial spider silks, whereas customized spidroins make it possible that artificial spider silk-like fibers can be spun from biological buffers at different pHs. To our knowledge, this study is the first to design customized FlSps with employing the NTD, repetitive region, and CTD from the same spidroin and same spider species. The customized FlSps were able to form artificial spider silk-like fibers with outstanding mechanical strength and decent extensibility from “spidroin” solutions with a rather low concentration (∼1 mg/mL) at pH 8.0. Our results shed light on the fact that FlSp terminal domains are essential for silk formation, and the new customized FlSp-derived silk-like fibers hold application potentials as novel biomaterials.

## Supporting Information Available

Amino acid composition and hydropathy profile of the repetitive domain S from flagelliform spidroin; mechanical testing curves of 10 samples of each type of fibers; ATR-FTIR spectral decomposition in the amide I region of silk-like fibers; primer for cloning; and calculated content of the secondary structure of the silk-like silks (PDF)
