# Supplementary material for: Customized Flagelliform Spidroins Form Spider Silk-like Fibers at pH 8.0 with Outstanding Tensile Strength
Source: ACS Biomater Sci Eng. 2021 Dec 15;8(1):119–27. doi: 10.1021/acsbiomaterials.1c01354 (PMC8753598; doi:10.1021/acsbiomaterials.1c01354)
Supplement: Supplementary file 1 — ab1c01354_si_001.pdf [file ab1c01354_si_001.pdf]

Customized flagelliform spidroins form spider silk-like fibers at pH 8.0 with outstanding tensile strength

Xue Li[1,2], Xingmei Qi[3], Yu-ming Cai[4], Yuan Sun[2], Rui Wen[2], Rui Zhang[5], Jan Johansson[6], Qing Meng[2,*], Gefei Chen[6,*]

[1]Department of Medical Ultrasound, Shanghai Tenth People's Hospital, Ultrasound Research and Education Institute, Tongji University Cancer Center, Shanghai Engineering Research Center of Ultrasound Diagnosis and Treatment, Tongji University School of Medicine, 200092, Shanghai, China.

[2]Institute of Biological Sciences and Biotechnology, Donghua University, 201620, Shanghai, China.

[3]The Jiangsu Key Laboratory of Infection and Immunity, Institutes of Biology and Medical Sciences, Soochow University, Suzhou, 215123, China

[4]Institute for life sciences, University of Southampton, SO17 1BJ, Southampton Hampshire, UK.

[5]Department of Pulmonary Circulation, Shanghai Pulmonary Hospital, Tongji University School of Medicine, Shanghai, 200433, China

[6]Department of Biosciences and Nutrition, Karolinska Institutet, 14157, Huddinge, Sweden.

*Corresponding author: Qing Meng (e-mail: mengqing@dhu.edu.cn) and Gefei Chen (e-mail: gefei.chen@ki.se)

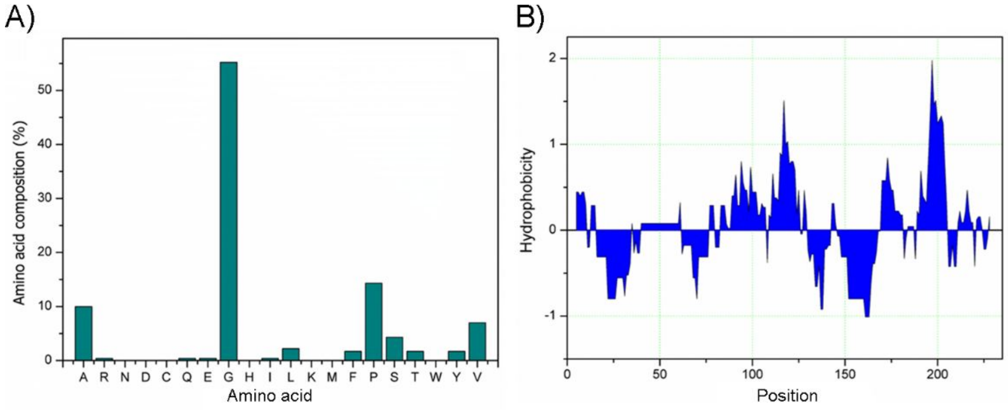

Figure S1 Amino acid composition and hydropathy profile of the repetitive domain S from flagelliform spidroin. A) Amino acid composition of single repetitive region S. Amino acids are shown with one letter. B) Hydrophobicity analysis of the S domain, which was predicted by Kyte & Doolittle calculation (https://web.expasy.org/protscale/).

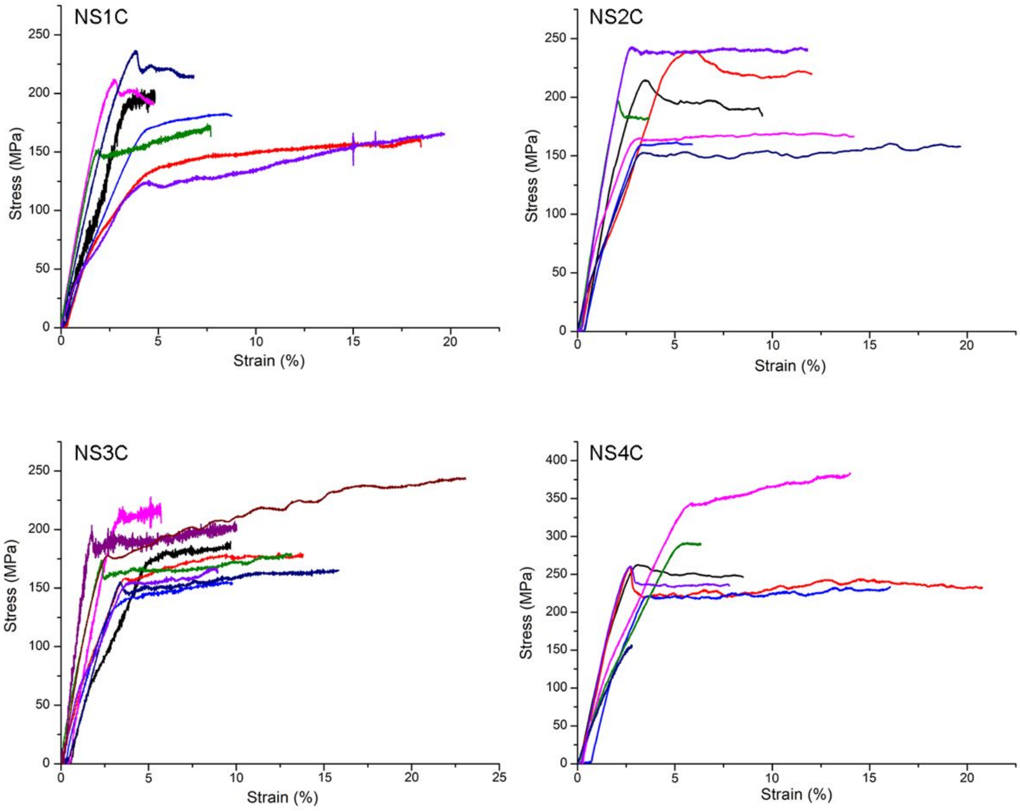

Figure S2 Mechanical testing curves of ten samples of each type fibers.

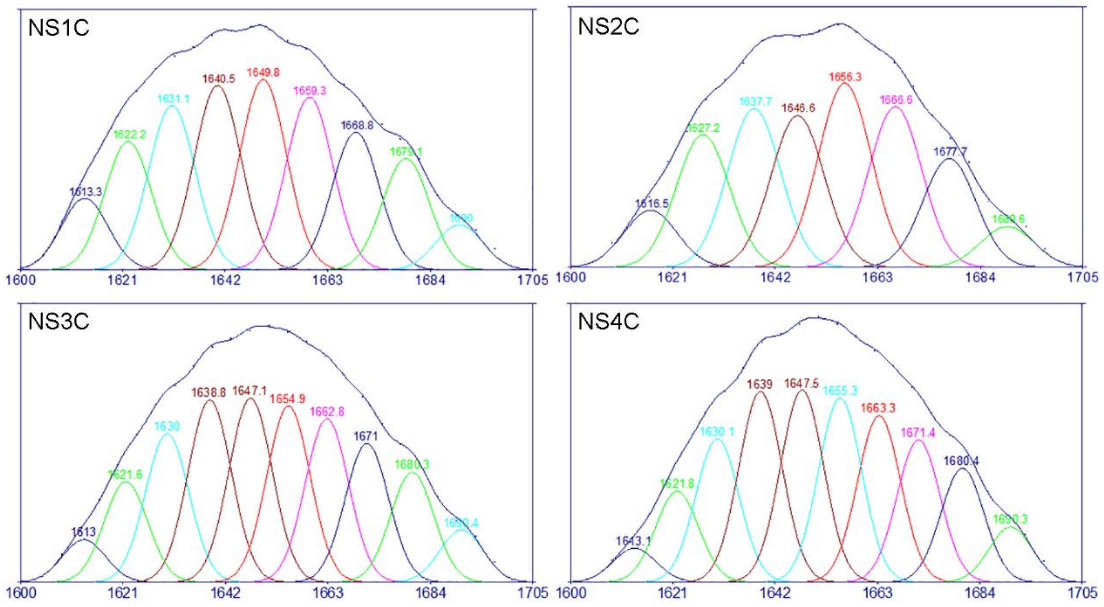

Figure S3 ATR-FIRT spectral decomposition in the amide I region of silk-like fibers.

Table S1 Primer for cloning.

Primer	DNA Sequence (5'-3')
NTDP1	agctGGATCCAGGGGCATTCATGTCTC
NTDP2	agctGGTCTCGGCGCCCTCGTTAAACTGCTCTTGAGAGAATAG
CTDP1	agctACCTGCGGTGGCGCTGGTGGTCAGCCGTCTGGTGGTG
CTDP2	agctCTCGAGTTAATATCCAAAGCATCTTCGGAC

Nucleotides in lowercase are designed for improving digestion efficiency

Table S2 The calculated content of the secondary structure of the silk-like silks.

	NS1C	NS2C	NS3C	NS4C
β-sheet	43.23±3.46	36.75±2.32	37.71±2.38	35.27±0.29
random coil	15.60±1.85	14.02±1.08	16.29±1.89	15.17±2.65
α-helix	14.14±1.74	16.06±2.11	15.23±1.54	14.67±1.36
β-turn	27.04±2.37	33.18±2.46	30.76±4.53	34.89±3.97
